# Dr. Giraldes on Organs for the Secretion of Sweat

**Published:** 1842-10-01

**Authors:** 


*Organs for the Secretion of Sweat.* By Dr. Giraldes.—The organs which secrete the sweat, which were first pointed out by Breschet, and afterwards described by Purkinje, Gurlt, Wagner, Serres, Arnold, Madden, and others, Dr. Giraldes has studied more particularly, and added a few additional facts. These glands exist in great numbers in the palm of the hand, and in the soles of the feet, in which situations they are very numerous, and being well developed, are more easily examined than elsewhere. They exist also, and are of tolerable size, in all the spots covered with hair, as the arm-pits, perineum, scalp, &c. They are also seen over the whole surface of the derma; but are there extremely small, almost rudimentary. These organs are composed, according to Dr. Giraldes, not of simple canals divided at their extremity, but of a straight canal, which pierces through the whole thickness of the derma, and is imbedded in the fatty layer beneath it. Sometimes they penetrate this fatty layer to a very considerable depth; at the extremities of the fingers they even seem to traverse it completely. Arrived at this point, these canals sometimes dichotomize; in general, however, they remain simple, and roll on themselves, so as to form a small button-shaped body. These convolutions are sometimes all on the same plane; at other times so as to form a spheroidal appearance. Dr. Giraldes states that he has shown this structure to Professor Serres.—*Comptes Rendus des Séances de l'Academie des Sciences: and Edinburgh Medical and Surgical Journal.*

